# A Case of Typhoid Fever with Hepatic Granulomas and Enteritis

**DOI:** 10.1155/2015/745461

**Published:** 2015-01-28

**Authors:** Shraddha Narechania, Marc Duran, Vidhya Karivedu, K. V. Gopalakrishna

**Affiliations:** ^1^Fairview Hospital, Cleveland, OH 44111, USA; ^2^Internal Medicine Residency, Atlanta Medical Center, 303 Parkway Drive NE, Atlanta, GA 30312, USA; ^3^Internal Medicine Residency Program, Fairview Hospital, Cleveland, OH 44111, USA

## Abstract

The common histopathologic hepatic manifestations in patients infected with *Salmonella* include cloudy swelling and balloon degeneration with vacuolation of the hepatocytes and steatosis. Hepatic granulomas are a very rare finding, so far reported in very few cases. We report a 64-year-old patient with *Salmonella* enteritis who was found to have multiple 1.4 to 1.6 cm hypoechoic liver masses on ultrasound of the abdomen which on biopsy revealed hepatic granulomas. This case highlights the importance of keeping the differential diagnosis of *Salmonella typhi* (*S. typhi*) in mind in a patient with hepatic granulomas.

## 1. Introduction

Typhoid is an infection seen in developing countries caused by the bacterium* S. typhi*. It is rare in developed countries like United States. As per CDC, approximately 5700 cases of* S. typhi* occur in the United States every year. Out of these most cases (75%) are acquired while travelling internationally. Route of transmission is through feck-oral route. Infection with* S. typhi* is known to cause hepatitis with findings of focal hepatocellular necrosis and nonspecific inflammation on liver biopsy. Granulomas are extremely rare to find in hepatitis caused by* S. typhi* and have been reported only in a few cases till date.

*Objective*. To describe a rare case of typhoid fever with granulomas in the liver.

## 2. Case Report

A 64-year-old Cambodian female presented with a two-week history of abdominal pain, bloody diarrhea, nausea, poor oral intake, and mild fever. Her past medical history included arterial hypertension, hypercholesterolemia, and type two diabetes mellitus. There was no history of inflammatory bowel disease or colon cancer in her family. Her medications included metoprolol, simvastatin, and metformin. She had travelled to Cambodia recently but had no exposure to sick contacts. Clinical exam presented an ill, pale appearing woman with blood pressure 124/60 mm Hg, heart rate 115 beats per minute, respiratory rate 16 per minute, and temperature 99.7°C. She later developed a temperature max of 39.4°C (103°F). She had right-upper quadrant and epigastric tenderness. No hepatosplenomegaly could be appreciated and Murphy's sign was negative. Laboratory studies showed white blood cell count of 7640/*μ*L, hemoglobin of 10.8 gm/dL, and hematocrit of 31.1% with serum potassium of 3.1 mmol/L and serum calcium 7.9 mg/dL. Liver profile showed total bilirubin of 0.4 mg/dL, total protein of 6.4 g/dL, albumin of 2.9 g/dL, AST of 218 U/L, ALT of 131 U/L, and alkaline phosphatase of 209 U/L. Blood, urine, and stool specimens were collected and sent for culture. Stool studies were negative for* Shigella*,* Yersinia*,* Campylobacter*,* E. coli*, and* C. diff*. Blood cultures were taken and all 4 bottles of the 2 sets grew* S. typhi*. Ultrasonography showed multiple hypoechoic liver masses of 14 to 16 mm in size along with gallbladder sludge and otherwise normal biliary tree. Computed tomography of the abdomen showed a homogenous enhancement without any focal abnormality in the liver and wall thickening involving the terminal ileum without surrounding inflammatory changes. It was reported that the focal masses seen on ultrasound were thought to be atypical hemangiomas. Magnetic resonance imaging showed during the arterial phase innumerable hyperenhancing masses throughout the liver. A hepatobiliary iminodiacetic acid (HIDA) scan reported normal hepatocellular function, patent cystic duct, common bile duct, and sphincter of Oddi. Upper endoscopy showed diffuse stomach erythema with small 4 × 4 mm ulcer in the antrum and a superficial ulcer in the duodenal bulb along with a deep 1 × 1 cm ulcer in the apex of the bulb that were both cauterized. Colonoscopy showed deep clean-based ulcerations in the cecum and ascending colon and scattered superficial ulcerations in the transverse colon. Gastric biopsy reported minimal superficial chronic gastritis without activity. Duodenal biopsy reported focal active duodenitis. Colon biopsy reported focal active enteritis in the terminal ileum with patchy active colitis in the cecum ascending and transverse colon. There was no evidence of viral inclusions, dysplasia, or malignancy. A focal crypt rupture granuloma was seen in the transverse colon but no true mucosal granulomas were seen. Liver biopsy (A, B) reported normal lobular architecture without fibrosis. Bile ducts appeared normal in number and morphology and hepatic vasculature was unremarkable. Portal tracts show a mixed inflammatory infiltrate consisting of lymphocytes, histiocytes, and occasional scattered plasma cells and neutrophils. Numerous suppurative granulomas were scattered throughout the hepatic lobules. The patient initially received IV Ciprofloxacin 400 mg every 12 hours and IV Ceftriaxone 2 gm every 24 hours for a total of 2 weeks each while she was in the hospital and was eventually discharged on IV Ceftriaxone 2 grams every 24 hours for a total of 6 weeks.

## 3. Discussion

**Figure 1:**
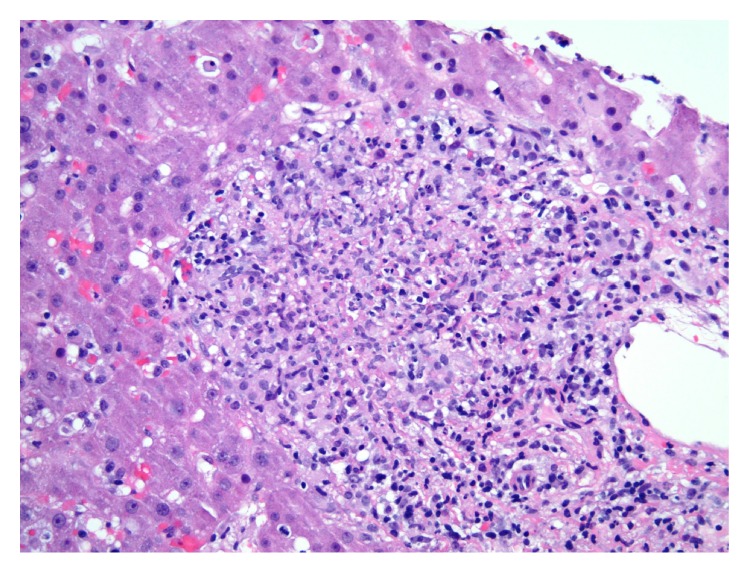
H&E staining of biopsy specimen showing hepatic granuloma in 20x magnification.

**Figure 2:**
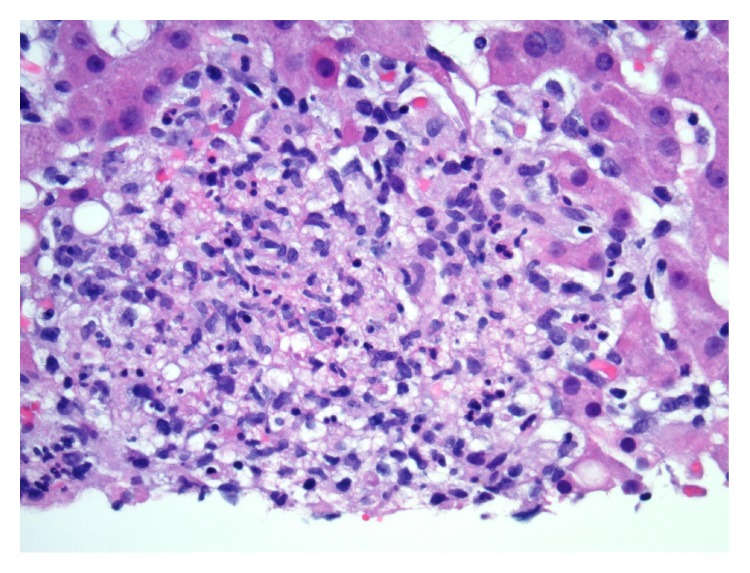
H&E staining of biopsy specimen showing hepatic granuloma in 40x magnification.

The etiology of hepatic granulomas can be divided into infectious, noninfectious (immune), foreign body reactions, drugs, and neoplasia. Well-reported causes of hepatic granulomas in developed countries include sarcoidosis, tuberculosis, primary biliary cirrhosis, drug reactions, and malignancy [1]. Our patient was suffering from typhoid enteritis but her clinical status was transformed to typhoid fever and hepatitis due to her immunocompromising risk factor of diabetes mellitus. This case highlights and further describes the manifestations that can be associated with* S. typhi* and typhoid fever, specifically hepatic and GI involvement. Association between typhoid and systemic granulomas has been reported in very few cases. To our knowledge, only 5 cases of hepatic granulomas have been reported so far in the English language literature. Pais [2] reported the first 2 cases of typhoid hepatitis with hepatic granulomas in 1984. The third case of typhoid associated hepatic granulomas was reported by Satti et al. [3] in 1990. They reviewed 59 cases of patients with hepatic granulomas out of which 1 case was due to* S. typhi*. Mert et al. [4] reported 2 more cases of typhoid fever in 2004 with hepatic granulomas who also had splenic and bone marrow granulomas, respectively. The first case showed noncaseating granulomas and the second one showed a single microgranuloma on liver biopsy. Typhoid cases with granulomas in other sites like ileum, colon, ovaries, and breast tissue have also been reported. The present case would be the sixth case reported of a patient with typhoid having hepatic granulomas (Figures 1 and 2). In all the previously reported cases, the biopsy showed very few or one granuloma which was nonnecrotizing. Our case showed multiple suppurative necrotizing granulomas. In our case, the AFB stain done on Liver biopsy samples was negative. Sarcoidosis can also present as hepatic granulomas but our patient did not have any other manifestations suggestive of sarcoidosis. Crohn's disease rarely causes hepatic granulomas; however, in our patient, positive blood cultures for* S. typhi* and recent travel to an endemic region support the diagnosis of Salmonella infection. Mainstay of diagnosis for* S. typhi* infection is with blood cultures. Histologic findings of typhoid fever include macrophage aggregates found commonly in the intestines, mesenteric lymph nodes, spleen, liver, and bone marrow. Liver biopsy in these individuals shows cloudy swelling and balloon degeneration with vacuolation of the hepatocytes and steatosis.

Hepatic granulomas have been reported rarely, only in 5 cases until now. The present case is the 6th case reported. It is hard to say if this is really a rare manifestation of* S. typhi* or if it is under reported.* S. typhi* is an infection seen mainly in developing countries whereas our patient was diagnosed in a developed country where extensive measures are undertaken to characterize a patient's disease.
